# Investigation of Multi-Factor Coupled Aging Mechanisms and Rheological Performance Prediction of Asphalt in Diverse Climatic Regions

**DOI:** 10.3390/ma19143127

**Published:** 2026-07-21

**Authors:** Hong Xu, Shanglin Song, Fangxia Wang, Xiaolei Wu, Yang Luo, Xiaoyan Ma, Ningyuan Meng, Tianyu Wu

**Affiliations:** 1 Gansu Provincial Highway Development Group Co., Ltd., Lanzhou 730070, China; 13919083809@163.com (H.X.); 18009425159@163.com (Y.L.); 2 Scientific Observation and Research Base of Transport Industry of Long Term Performance of Highway Infrastructure in Northwest Cold and Arid Regions, Dunhuang 736200, China; 18193106299@163.com; 3 Qinzhou Highway Section, Tianshui Highway Development Center of Gansu Province, Tianshui 741000, China; 13389481107@163.com; 4 School of Materials Science and Engineering, Chang’an University, Xi’an 710064, China

**Keywords:** asphalt binder, aging mechanism, thermal-oxidative aging, coupled environmental aging, rheological properties, climatic regions

## Abstract

Aging of asphalt pavements is a complex, multi-scale degradative process driven by the synergistic effects of various environmental stressors. Traditional laboratory-accelerated aging protocols often employ static parameters that fail to accurately replicate dynamic, region-specific climatic conditions. To bridge the gap between laboratory simulations and actual field performance, this study investigates the aging behaviors of base binder and SBS-modified binder under multi-factor coupled environmental conditions. Field observations were conducted across six distinct climatic regions in Gansu Province, alongside an indoor second-order orthogonal regression composite design that evaluated the interactive effects of temperature, ultraviolet (UV) radiation, humidity, and aging time. Rheological evaluations revealed that for the base binder, the synergistic coupling of UV radiation, elevated temperatures, and high humidity significantly accelerates oxidative hardening and embrittlement far beyond the impact of any single factor. Conversely, SBS-modified binder demonstrated a non-linear, U-shaped rheological response governed by a competitive mechanism between UV/thermal-induced polymer scission and moisture/time-driven matrix oxidation. Fourier Transform Infrared (FT-IR) spectroscopy corroborated these macroscopic findings at the molecular level, tracking the simultaneous evolution of carbonyl and sulfoxide indices alongside the degradation of the polybutadiene segments in the modified binder. Ultimately, a quadratic polynomial regression model was established to precisely correlate natural field aging with equivalent indoor accelerated aging times based on specific regional climatic data.

## 1. Introduction

Asphalt pavements constitute one of the most widely used forms of paved road infrastructure worldwide. In the United States, for example, asphalt-surfaced pavements are predominant; according to an FHWA-based analysis of pavement surface-type data, approximately 94% of paved road miles are surfaced with asphalt [1]. This extensive use means that even incremental improvements in asphalt pavement durability can have substantial implications for material conservation, maintenance demand, and environmental sustainability. When pavements age prematurely, repeated maintenance and rehabilitation consume additional binder, aggregates, energy, and financial resources, while also increasing traffic disruption and associated environmental burdens. Thus, understanding the mechanisms of binder aging is not only a materials-science problem, but also a key pathway to enhancing pavement sustainability and resource efficiency. To avoid the ambiguity that arises because “asphalt” can mean either the hot-mix mixture (Europe) or the neat asphalt cement (this study), this manuscript consistently uses “binder” to refer exclusively to the pure bituminous material.

Its widespread application is attributed to an optimized balance of engineering properties, including superior driving safety, exceptional surface smoothness, high structural strength, and effective noise attenuation [2]. These characteristics ensure not only the comfort of road users but also the operational efficiency of logistics. However, binder is an organic hydrocarbon material, making it inherently susceptible to environmental stressors. During its extended service life, binders inevitably undergo a phenomenon known as aging. This is a coupled chemo-rheological process involving oxidation, volatilization, molecular association, and redistribution of binder fractions [3,4,5,6]. At the molecular level, aging involves the reorganization of the binder’s internal structure and the redistribution of its micro-components. Macroscopically, these changes manifest as the deterioration of rheological performance, characterized by increased stiffness and reduced stress relaxation capacity. Such impairment poses significant risks to road traffic safety, leads to premature pavement failure, and imposes substantial economic burdens on pavement maintenance and rehabilitation. Therefore, achieving a holistic understanding of the environmental factors driving binder performance degradation is of paramount importance for the sustainable development of road infrastructure.

Existing research consistently identifies temperature as the most critical kinetic driver of binder aging. Elevated temperature accelerates oxygen diffusion and promotes the formation of carbonyl and sulfoxide groups, while long-term oxidation increases binder viscosity and changes the balance between maltenes and asphaltenes [7,8]. Thus, binder aging is fundamentally defined as a typical slow oxidation process, within which thermal-oxidative aging represents the primary internal mechanism. Studies have indicated that during periods of peak solar radiation, the surface temperature of an asphalt pavement can reach approximately twice that of the ambient air temperature [9]. This localized heat accumulation intensifies molecular thermal agitation, providing the requisite activation energy for oxidation and leading to profound alterations in the binder’s chemical composition. When temperatures exceed 100 °C, the hydrocarbons within the binder undergo complex polycondensation and polymerization. These processes result in the formation of increasingly larger macromolecular clusters, which impairs the stability of the colloidal system and increases its overall heterogeneity [7]. Studies on polymer-modified asphalt further show that thermal aging involves two competing processes: oxidative hardening of the base binder and degradation of polymer networks such as SBS [10,11]. For instance, Wang et al. [10] conducted a comparative analysis of short-term and long-term aging using the Rolling Thin-Film Oven Test (RTFOT) and Pressure Aging Vessel (PAV). Their results demonstrated that during the RTFOT stage, the high-temperature environment primarily triggers the thermal degradation of polymer modifiers, such as Styrene-Butadiene-Styrene (SBS), leading to a notable decrease in the styrene-butadiene ratio. Conversely, during the PAV stage, sustained high temperatures and pressures accelerate the oxidation of the base binder matrix itself, resulting in a significant increase in the Carbonyl Index. This distinction underscores that temperature drives different aging modes depending on the duration and intensity of exposure. Furthermore, Poulikakos et al. [12] highlighted a dual effect: while high-temperature aging may improve the stability of asphalt mortars in the short term by increasing stiffness (rutting resistance), it concurrently shifts the brittle point toward higher temperatures. This increased brittleness severely weakens the fatigue resistance and low-temperature crack resistance of the material, making the pavement prone to thermal cracking [13]. This densified microstructure hardens the binder’s macroscopic rheological performance: The complex modulus (G*) rises while the phase angle (δ) declines. Although such stiffening may temporarily enhance resistance to high-temperature rutting, it reduces stress relaxation capacity and shifts the material toward a more brittle state, thereby weakening fatigue resistance and low-temperature cracking resistance [14].

Another critical internal mechanism is ultraviolet (UV) aging, where the intensity and duration of solar radiation become the dominant inducing factors. UV radiation carries significantly higher energy than thermal energy and is capable of directly cleaving the chemical bonds (C–C, C–H) within binder molecules. This process initiates free-radical chain reactions, leading to the formation of a highly oxidized, brittle layer on the pavement surface [15,16,17]. Despite its high energy, UV radiation has a limited penetration depth. Zeng [18] utilized Ultraviolet Spectrophotometer Tests (UST) and the delamination method to quantify this effect, revealing that direct UV-induced aging is confined to a depth of approximately 4.5 μm. However, the aging effects can migrate deeper into the binder film through surface diffusion and secondary reactions. The synergistic effect of the penetration depth of ultraviolet rays and surface diffusion controls the spatial gradient of aging [17,19]. Celauro et al. [20] further studied the coupling effect of RTFOT short-term aging and UVB irradiation on neat binders and SBS-modified binders. Their rheological and FTIR test results demonstrated that UV radiation not only promotes the generation of oxygen-containing functional groups in neat binders but also causes the fracture of polymer chains in SBS-modified systems. Their findings also indicated that the SBS modifier can significantly enhance the overall anti-UV aging performance of binders, thereby providing an important reference for the application of modified binders in high-UV regions. In addition, environmental factors have a non-negligible regulatory effect on the above aging behaviors. Garcia Mainieri and Al-Qadi [21] further showed that controlled moisture and temperature conditions can change UV aging behavior, indicating that UV aging should not be simplified to dry radiation aging alone.

Field and surface-characterization evidence confirms that photo-oxidation and thermal oxidation interact strongly. Li et al. [22] analyzed different pavement sections—including modified, unmodified, and tunnel pavements (the latter shielded from UV)—and found that the Carbonyl Index (*I_C_*_=_*_O_*) in the top layer of the exposed sections was significantly higher than that of the tunnel sections. This directly confirms that UV radiation is a primary accelerator of surface-layer binder aging. Yang et al. [23] showed that coupled thermal-UV aging changes surface morphology, oxygen-containing functional groups, surface polarity, and rheological response. These findings are consistent with broader chemo-rheological assessments in which FTIR indices, microscopic morphology, and dynamic shear parameters jointly indicate the development of the aged binder structure [5]. For SBS-modified binder, the impact is even more structural. Yu [24] observed that UV irradiation destroys the three-dimensional cross-linked network formed by the SBS modifier, essentially “unzipping” the polymer chains and causing a rapid loss of the elastic recovery properties that the modifier was intended to provide. Consequently, continuous UV exposure not only ages the matrix but also deactivates the modification system, leading to surface micro-cracking and raveling.

The progressive aging of asphalt pavements leads to a gradual deterioration of their viscoelastic properties, inevitably leading to material failure. Wang et al. [25] demonstrated that prolonged aging duration leads to a monotonic enhancement in the high-temperature stability of binders, accompanied by a steady increase in creep stiffness. However, this gain in stiffness is coupled with a marked elevation of the brittle point, rendering asphalt mortar highly susceptible to cracking under declining temperature gradients. Li et al. [26] found that under the synergistic influence of thermal and photo-oxidative stressors, extended aging results in a significant accumulation of asphaltenes at the expense of aromatics. This fundamental shift in the SARA fractions underscores the mechanism by which deep aging impairs the low-temperature fracture resistance of binders. Similarly, Im et al. [27] confirmed that aging duration dictates the mechanical response of binders, where the escalation of tensile strength and stiffness is counterbalanced by a catastrophic reduction in crack resistance. From a fracture mechanics perspective, Mansourian and Amiri [28] further established a strong correlation between the fracture toughness in Mode I (K_IC_) and the conventional bending beam rheometer (BBR) parameters, proposing that K_IC_ can serve as an effective indicator to evaluate the low-temperature performance of aged binders. This fracture-mechanics-based approach is consistent with the performance evolution law observed in the present study.

While high temperature and UV are often the focus of aging studies, the role of humidity and moisture cannot be overlooked. Asphalt pavements exposed to high-humidity environments or frequent precipitation are prone to peeling, stripping, and accelerated cracking. The influence of moisture is expressed through the complex interaction between water molecules and the polar groups within the binder [29]. Water acts as a solvent that facilitates the leaching or dissolution of certain polar chemical constituents, which in turn alters the balance of the SARA fractions. To clarify these micro-mechanisms, Hung et al. [30] employed Atomic Force Microscopy (AFM) and Fourier Transform Infrared Spectroscopy (FTIR), discovering that water exposure triggers a drastic evolution in the binder’s surface morphology (topological structure). The concentration of surface polar components increases under moisture exposure, leading to premature adhesive failure between the binder and the aggregate. Similarly, Noguera et al. [31] found that the presence of water promotes the dissolution of resins and aromatics, shifting the binder toward a more asphaltene-rich, brittle state. Recent studies by Ma et al. [32] using specialized environmental chambers confirmed that higher humidity levels correlate with an increase in macromolecular content and a decrease in small molecules, further accelerating the oxidative aging of the matrix and the degradation of SBS polymers.

In a real-world service environment, the binder is never subjected to a single aging factor in isolation. Instead, it experiences the synergistic coupling effect of temperature, UV radiation, moisture, and oxygen. In high-altitude regions like the Qinghai–Tibet Plateau or the Yunnan–Guizhou Plateau, these factors are present in extreme combinations: Intense UV radiation occurs alongside drastic diurnal temperature swings and frequent cycles of rain and snow. This multi-factor synergy often leads to accelerated pavement deterioration that exceeds the sum of individual factors [33].

Research by Li et al. [34] on high-viscosity binders demonstrated that the coupling of heat, UV, and acid rain has a far more profound impact on viscoelasticity than any single stressor. Similarly, Menapace et al. [35] emphasized that the combined action of UV and moisture increases brittleness and surface cracking in a way that single-factor laboratory tests cannot replicate. In fact, studies on reactive oxygen species have shown that realistic atmospheric aging may involve ozone, nitrogen oxides, and moisture-related chemistry in addition to molecular oxygen [36]. Yang et al. [37] further noted that while UV provides the energy to accelerate thermal-oxidative reactions, the presence of water can paradoxically change the activation energy of these reactions, complicating the prediction of aging rates. These results suggest that humidity should not be treated only as a mechanical stripping factor; it can also participate in the chemical and microstructural evolution of asphalt binder, especially when coupled with reactive oxygen species or direct water exposure [38].

Despite these insights, a significant research gap remains: the vast majority of current binder aging research is based on laboratory-accelerated aging protocols (e.g., TFOT, RTFOT, PAV) that use static, idealized parameters. These tests often fail to incorporate the dynamic service parameters of the real environment, such as fluctuating humidity and varying UV intensities. Consequently, laboratory results frequently struggle to accurately reproduce the mechanisms and performance evolution of natural aging, leading to discrepancies between predicted and actual pavement life. Beyond the mismatch between static laboratory conditions and real dynamic service environments, only a limited number of studies have connected indoor multi-factor aging results with field observations from climatically distinct regions [38,39]. A model that links laboratory-controlled environmental variables with field service conditions is therefore needed to improve the transferability of accelerated aging results.

To bridge the gap between laboratory simulations and field performance, this study adopts a comprehensive approach by integrating field observation with advanced statistical modeling. Five distinct climatic regions in Gansu Province, characterized by diverse temperature and humidity profiles, were selected as field observation points. Two representative materials, 90# base binder and SBS-modified binder, were chosen for investigation. The primary objectives of this research are: (1) to develop a quadratic orthogonal regression model that quantifies the coupling influence of temperature, UV radiation, aging time, and humidity on the rheological performance of binders, where the complex modulus is measured via a Dynamic Shear Rheometer (DSR); (2) to utilize FTIR spectroscopy to track the evolution of functional groups at different depths and aging stages, thereby revealing the underlying molecular-level aging mechanisms; and (3) to establish an accelerated aging prediction equation grounded in indoor simulation tests. By inputting the actual environmental parameters from six specific observation points, the model aims to accurately simulate and predict regional binder aging. This research will provide a robust framework for predicting binder aging under complex climatic conditions, offering valuable theoretical support for the selection of binder materials and the optimization of pavement maintenance strategies in varied geographical regions.

**Figure 1:**
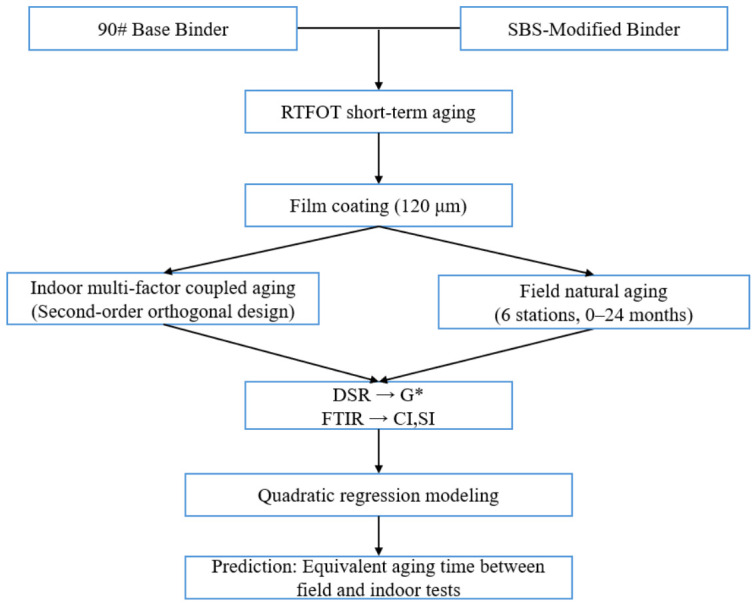
Experimental flowchart of the study.

The experimental flowchart designed in this study is summarized in Figure 1.

## 2. Experimental and Analytical Methods

### 2.1. Materials

The base binder used in this study is a 90# penetration-grade asphalt cement conforming to the Chinese specification JTG E20-2011 [40]. The designation “90#” indicates a penetration range of 80–100 dmm (0.1 mm) at 25 °C. The key properties of the 90# base binder are summarized in Table 1. All tested properties meet the specification requirements.

### 2.2. Preparation of Binder Film Specimens

The thickness of the binder specimen is a critical parameter in ultraviolet (UV) aging experiments, as it directly governs the magnitude and uniformity of photo-oxidative degradation. Current literature lacks a standardized thickness for UV exposure, with reported values ranging from 100 μm to over 3.2 mm. However, given that the penetration depth of UV radiation into bituminous materials is highly limited, thicker samples often develop a significant aging gradient where the unaged bulk material dilutes the observed rheological changes. Consequently, utilizing thinner binder films is essential to ensure that the experimental results accurately reflect the true extent of photochemical degradation and to enhance the sensitivity of the binder to environmental stressors.

**Figure 2:**
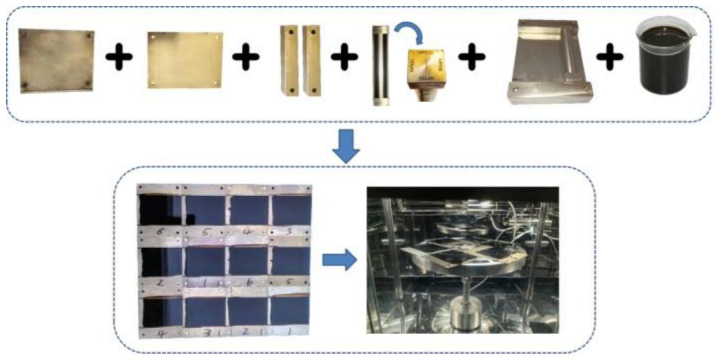
Binder film specimen preparation and subsequent accelerated UV aging test. Notes: (**Top frame**): Components of a self-made precision film coater; (**Bottom left**): Binder films with four designed thicknesses (30/60/90/120 μm); (**Bottom right**): Specimen rack placed in the UV aging chamber. All binders were pre-treated by RTFOT, and 120 μm films were adopted for formal tests.

To achieve high-precision control over specimen geometry and ensure reproducibility, a custom-designed film coating apparatus was developed in this study. The device features micro-scale adjustment modules that allow for the calibrated preparation of films with thicknesses of 30 μm, 60 μm, 90 μm, and 120 μm by maintaining a uniform gap between the precision-ground carrier plate and the coating track. The experimental protocol involved subjecting the virgin binder to the RTFOT to simulate short-term construction aging, followed by the preparation of the test films using the coating device. For the accelerated UV aging tests, a target thickness of 120 μm was selected as an optimized balance between maximizing UV exposure sensitivity and ensuring sufficient material integrity for subsequent rheological characterization. The preparation process and UV aging setup are shown in Figure 2.

### 2.3. Field Exposure and Natural Aging Procedure

A network of six outdoor exposure stations for natural aging was deployed throughout Gansu Province. These locations—Dunhuang (DH), Lanzhou (LZ), Tianshui (TS), Hezuo (HZ), Longnan (LN), and Tianzhu (TZ)—were strategically selected for their diverse environmental profiles. Specifically, DH features a classic hot and arid desert environment, whereas LZ experiences an inland continental climate within the semi-arid Loess Plateau transition area. In the southeastern part of the province, TS and LN offer warm and wet conditions characterized by elevated rainfall. Conversely, HZ presents a cold, damp plateau climate due to its high elevation, while TZ, situated on the periphery of the Qinghai–Tibet Plateau, is notable for its extreme altitude, freezing temperatures, and severe solar radiation. Collectively, these stations cover the primary climatic regions relevant to asphalt pavements. Every observation station was outfitted with automatic monitoring equipment to continuously track ambient temperature, humidity levels, and UV exposure. The exposure period covered 24 months, with sampling conducted every six months.

**Figure 3:**
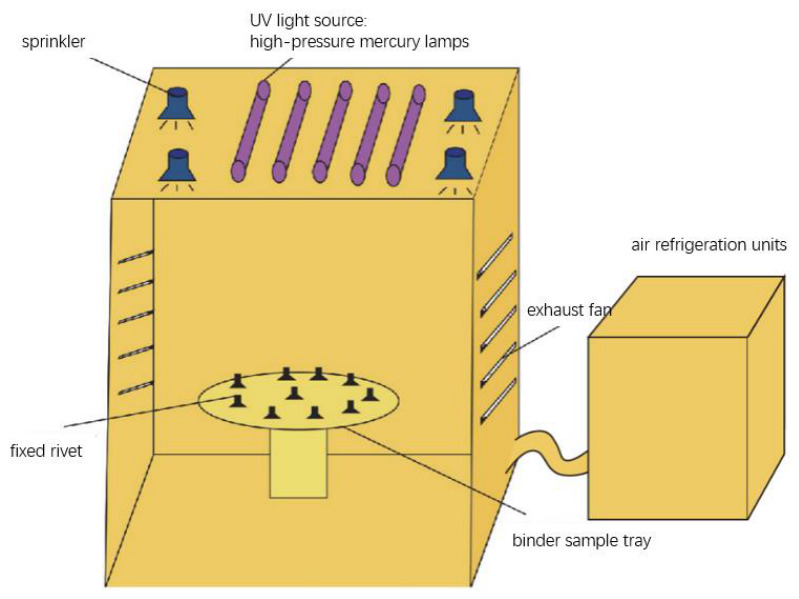
Schematic diagram of the internal structure of the binder UV aging chamber.

To replicate complex natural aging in the laboratory, a self-developed multi-factor coupled UV aging apparatus was used for indoor accelerated aging tests (Figure 3). This device is equipped with high-pressure mercury lamps, heating modules, air refrigeration units and atomizing sprinklers. The UV radiation intensity is adjusted by regulating the operating voltage of the mercury lamps; the top sprinkler controls internal humidity. Heating and refrigeration systems work cooperatively to achieve temperature increases and decreases, while side exhaust fans maintain uniform air circulation and stable temperature inside the chamber. With an independent refrigeration system, the apparatus can simulate various natural environmental conditions, including variable temperature, humidity and UV intensity.

Prior to indoor coupled aging, all specimens underwent standard RTFOT short-term aging to simulate construction-related aging. Two types of binders were tested in this study: 90# base binder and SBS-modified binder. For the field natural aging tests, asphalt binder films with a thickness of 3.2 mm were prepared, consistent with those used in the laboratory short-term aging tests. Three replicate specimens were tested for each binder to ensure the reliability of the results.

### 2.4. The Second-Order Orthogonal Regression Composite Design

The second-order orthogonal regression composite design is a sophisticated statistical framework employed to model and optimize complex processes involving multiple interdependent variables. Unlike traditional orthogonal array designs, this method integrates regression analysis to simultaneously evaluate the linear, interactive, and non-linear (quadratic) effects of factors on the response variable. By utilizing orthogonal arrays, the experimental factors are systematically varied in a balanced manner, effectively minimizing confounding effects and ensuring the independence of the estimated coefficients. The “second-order” capability is particularly critical for pavement materials research, as it allows for the accurate modeling of quadratic curvatures, which are prevalent in the performance evolution of binders and mixtures.

In this study, a quadratic regression model was fitted to the experimental data to express the response variable as a functional relationship of main effects, two-factor interactions, and quadratic terms. The mathematical structure enables the identification of optimal factor levels while capturing the intricate interdependencies within the system. Compared to conventional orthogonal designs, which are often restricted to linear main effects, the incorporation of quadratic terms significantly enhances the model’s fidelity in characterizing non-linear response surfaces. This makes the second-order design superior in identifying optimal factor settings that traditional methods might fail to capture.

**Figure 4:**
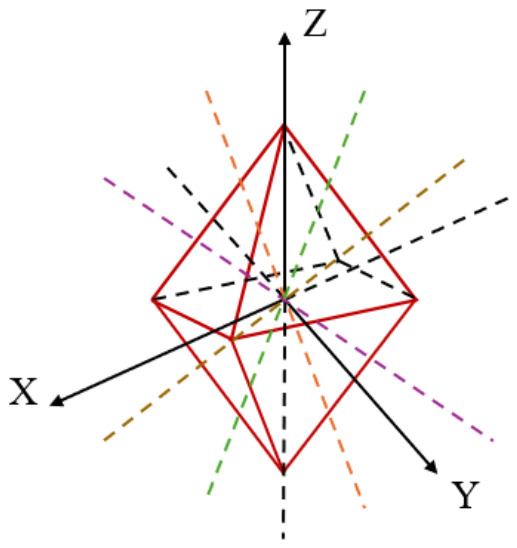
Schematic diagram of the second-order orthogonal regression composite design. The colored dashed lines indicate different coded-factor directions in the second-order orthogonal regression composite design, and the different colors are used to distinguish these directions.

From the perspective of experimental economy, the second-order orthogonal regression design offers higher resource efficiency than full factorial designs. It achieves high statistical power and reliable optimization insights with a significantly reduced number of experimental trials. This balance of precision and cost-effectiveness is particularly advantageous in material science and process engineering, where experimental resources and time are often constrained. The successful implementation of this design requires the precise identification of independent variables, the determination of center points (zero levels) for each treatment, and the strategic specification of variation ranges (coded levels) to ensure the design space adequately covers the potential optimal regions (Figure 4). For an experiment involving p factors, denoted as $z_{1},z_{2},z_{3},\cdots ,z_{p}$, each factor is assigned two levels: high and low. The high level of the j-th factor is represented by $z_{2j}$, and the low level by $z_{1j}$ (j=1, 2, ⋯, p). The zero level for each factor, $z_{0j}$, is defined as the arithmetic mean of the two levels:

(1) $$z_{0j}=\frac{z_{1j}+z_{2j}}{2}$$

The variation range for each factor is given by the difference between the high and low levels:

(2) $$\Delta_{j}=\frac{z_{1j}-z_{2j}}{2}$$

Before proceeding with the experimental design, it is crucial to determine the factors, their respective zero levels, and the variation ranges. In this study, a four-factor experimental design was implemented. The first factor is temperature, with a zero level of 20 °C and a variation range of 25 °C; the second factor is radiation intensity, with a zero level of 1000 watts and a variation range of 400 watts; the third factor is time, with a zero level of 5 h and a variation range of 2 h; and the fourth factor is humidity, with a zero level of 40% and a variation range of 20%. The experimental design is summarized in Table 2 and the results of the experimental design are shown in Table 3. It should be noted that all groups in Table 3 correspond exclusively to indoor accelerated aging tests, and these test conditions are not applied to field natural aging. Replicate samples were prepared for each experimental group.

### 2.5. Quadratic Polynomial Regression Model

The quadratic polynomial regression model serves as a robust statistical framework for characterizing and analyzing the functional relationship between a suite of independent variables (experimental factors) and a target dependent variable (response). Within the architecture of the second-order orthogonal regression composite design, this model is particularly efficacious in capturing both the linear trajectories and the non-linear curvatures of binder performance. By incorporating main effects, first-order interactions, and second-order (quadratic) terms, the model unveils the intricate interdependencies inherent in binder aging processes that traditional linear models often fail to detect.

In this study, the quadratic regression model is employed to construct a multi-dimensional response surface that shows the influence of environmental stressors on the binder’s properties. The general mathematical expression of the model is defined as follows:

(3) $$y=b_{0}+\sum_{i=1}^{m}b_{i}x_{i}+\sum_{i=1}^{m}b_{ii}{x_{i}}^{2}+\sum_{i=1}^{m}\sum_{j=1}^{m}b_{ij}x_{i}x_{j}$$

where y is the predicted response variable; $x_{i}$ represents the independent experimental factors; $b_{0}$ is the intercept (constant term); $b_{i}$ denotes the linear coefficients representing the main effects; $b_{ij}$ signifies the interaction coefficients between factors; and $b_{ii}$ represents the quadratic coefficients capturing the curvature.

This model provides a comprehensive understanding of how individual factors and their interactions affect the response, enabling the identification of factor levels that optimize the desired outcome. The inclusion of quadratic terms enables the model to capture non-linear relationships between factors and the response, providing a more accurate representation of real-world systems. It also allows for the identification of factor interactions, which are often crucial for understanding complex processes, and provides a framework for systematically optimizing multi-factor processes, improving efficiency and performance.

### 2.6. Rheological Testing (DSR)

**Figure 5:**
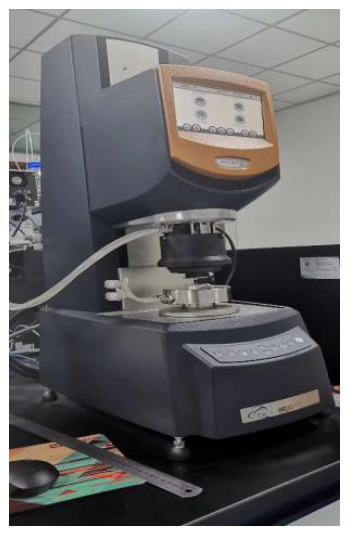
Dynamic shear rheometer.

To characterize the rheological properties of asphalt binders under different aging conditions, a TA Instruments AR20 dynamic shear rheometer (DSR; TA Instruments, New Castle, DE, USA) was employed (Figure 5). Sample geometries were standardized to a 20 mm diameter and 1 mm thickness. Initially, strain sweep tests were conducted to define the linear viscoelastic (LVE) limit. Since the lowest recorded LVE strain among all samples was 1.02%, a constant 1% shear strain was selected for subsequent evaluations. Final measurements of the complex modulus were then obtained via frequency sweeps conducted at 60 °C and an angular frequency of 1 rad/s.

All tested specimens were thin binder films prepared using a self-developed coating mould. Before DSR characterization, all virgin binders underwent standard RTFOT short-term thermal aging to simulate construction aging, followed by multi-factor coupled aging treatments involving temperature, UV radiation and humidity. The G* values of specimens treated only by RTFOT were defined as the initial zero baseline for comparison.

### 2.7. FTIR Test

**Figure 6:**
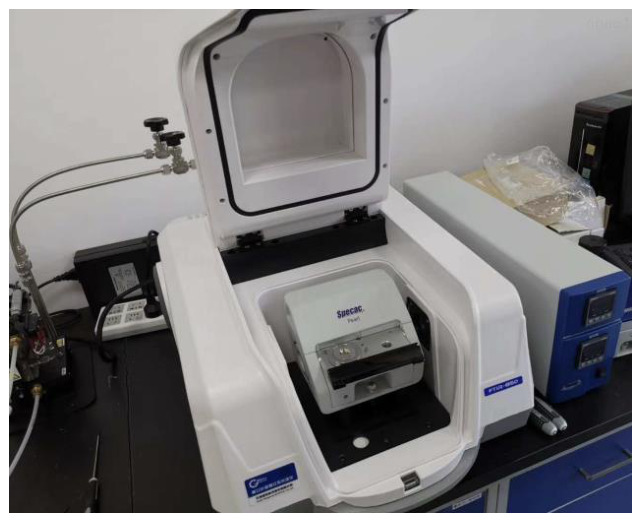
Fourier Transform Infrared Spectrometer (FTIR).

Fourier Transform Infrared Spectroscopy (FTIR) was employed to characterise the evolution of functional groups within the binders across different aging states and layers. In this study, a Nicolet iS50 spectrometer (Thermo Fisher Scientific, Waltham, MA, USA) equipped with an Attenuated Total Reflection (ATR) diamond crystal was utilized (Figure 6). The ATR mode was selected for its ability to directly measure the binder samples without the need for solvent dissolution, thereby preserving the original aging characteristics of the binder. The spectral data were acquired over a scanning range of 400~4000 cm^−1^. To ensure a high signal-to-noise ratio and reproducibility, each spectrum was obtained by averaging 32 scans. Prior to each test, the crystal surface was meticulously cleaned with ethanol, and a background scan was performed to eliminate environmental interference.

Binder aging is fundamentally characterized by the oxidation of specific reactive molecules, leading to the formation of carbonyl (*C*=*O*) and sulfoxide (*S*=*O*) groups. In the infrared spectra, these correspond to the characteristic absorption peaks at approximately 1700 cm^−1^ and 1030 cm^−1^, respectively. To quantitatively assess the degree of oxidation, the Carbonyl Index (*I_C_*_=_*_O_*) and Sulfoxide Index (*I_S_*_=_*_O_*) were calculated by normalizing the area of the characteristic peaks against the area of stable aliphatic C–H bending vibrations, as expressed in the following equations [41]:

(4) $$I_{C=O}=\frac{A_{1700}}{\sum A_{fingerpr\mathrm{int}}}$$

(5) $$I_{S=O}=\frac{A_{1030}}{\sum A_{fingerpr\mathrm{int}}}$$

where A_1700_ and A_1030_ represent the integrated areas of the carbonyl and sulfoxide peaks, respectively. $\sum A_{fingerpr\mathrm{int}}$ denotes the sum of the areas of the stable absorption peaks, which serve as an internal standard to account for variations in sample thickness.

By correlating the spatial evolution patterns of these oxygen-containing functional groups in samples from different depths, a quantitative assessment of the layered aging gradient was achieved. This analytical approach provides a molecular-scale bridge for explaining the macroscopic rheological hardening and embrittlement mechanisms of the asphalt pavement.

## 3. Results and Discussion

### 3.1. Impact of Environmental Factors on Base Binder Performance

#### 3.1.1. Effect of Single-Factor Influence on Base Binder Modulus in Natural Aging

**Figure 7:**
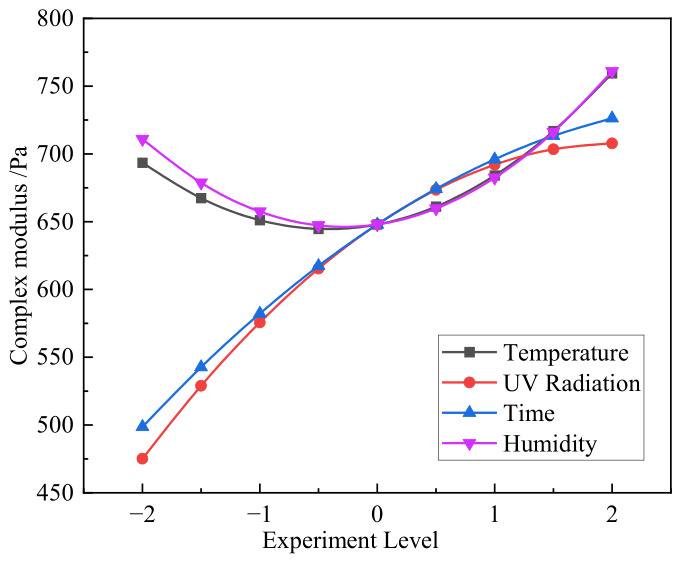
Effect of different experimental factors on the complex modulus of the base binder.

To evaluate the relative influence of environmental and operational variables on the rheological performance of the binder, a sensitivity analysis was performed based on the experimental results. As illustrated in Figure 7, the sensitivity analysis reveals distinct response patterns of the complex modulus to temperature, UV radiation, exposure time, and humidity across the coded experimental levels. Both UV radiation and exposure time exhibit a strong, monotonic positive correlation with G*, with UV radiation showing the most significant overall increase. This trend underscores a pronounced stiffening effect, indicating that photo-oxidation is a primary driver of material hardening. In contrast, temperature and humidity follow a non-linear, U-shaped trajectory. The complex modulus initially undergoes a marginal decline or stabilizes at lower levels before experiencing a sharp upswing as the factors reach the highest level. Notably, the dramatic rise in G* at high humidity levels suggests a threshold effect where moisture significantly accelerates material embrittlement.

The observed variations in the complex modulus reflect a complex interplay between oxidative hardening and physical softening mechanisms within the material matrix. The linear sensitivity to UV radiation and time suggests that prolonged exposure to high-intensity radiation facilitates molecular cross-linking and the formation of polar functional groups, thereby increasing the resistance to deformation. For temperature and humidity, the U-shaped response indicates a competition between softening effects and accelerated aging kinetics. At lower levels, the physical softening effect may partially offset the aging-induced hardening; however, at higher experimental levels, the accelerated chemical aging becomes the dominant mechanism, leading to a substantial increase in stiffness. These findings imply that while UV radiation remains the most consistent factor in promoting aging, extreme humidity conditions can act as a critical catalyst for rapid rheological degradation.

#### 3.1.2. Effect of Environmental Factor Interactions on Base Binder Modulus

**Figure 8:**
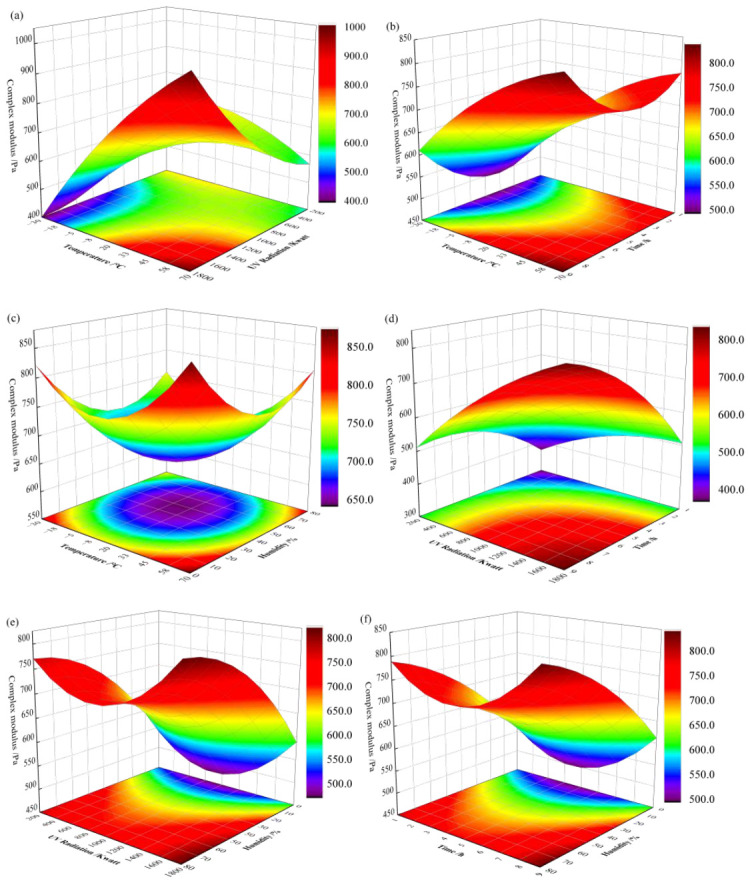
Interactive effects of environmental variables on the complex modulus of the base binder. (**a**) Temparature-UV radiation; (**b**) Temparature-Time; (**c**) Temparature-Humidity; (**d**) UV radiation-Time; (**e**) UV radiation-Humidity; (**f**) Time-humidity.

In real-world service conditions, factors such as temperature, UV radiation, and moisture do not act in isolation; rather, they exert a coupled influence that can significantly amplify or mitigate individual aging mechanisms. Evaluating the interactive effects between environmental stressors is critical for a comprehensive understanding of binder durability. Analyzing these interactions allows for the identification of synergistic or antagonistic relationships that single-factor analyses fail to capture. This holistic approach provides a more realistic representation of the binder’s rheological degradation, which is essential for developing predictive models and designing materials capable of withstanding complex climatic conditions. The 3D response surface plots in Figure 8 illustrate the specific synergistic effects of these variables on the complex modulus of the binder.

Figure 8a reveals a significant synergistic coupling between temperature and UV intensity. While temperature induces thermal softening, high-temperature conditions simultaneously accelerate oxidative kinetics. When UV radiation is introduced, photo-oxidation and molecular cross-linking are markedly enhanced, leading to a dramatic surge in G*. A threshold behavior is observed where G* escalates rapidly once both factors exceed moderate levels, indicating that the combined impact of intense sunlight and heat significantly exceeds the sum of their individual effects. As shown in Figure 8b, temperature and aging time work synergistically to promote oxidative hardening. Elevated temperatures increase the chemical reactivity of the binder matrix, facilitating faster oxygen uptake over time. Consequently, the slope of G* versus aging time becomes steeper at higher temperatures. At lower temperatures, the influence of time is less pronounced, whereas extreme conditions trigger a sharp rise in stiffness, reflecting high susceptibility to long-term oxidative degradation. Figure 8c demonstrates that humidity modulates the thermal aging process. While humidity may show minor stabilizing effects at lower levels, under high-temperature conditions, increased humidity facilitates oxygen diffusion into the binder matrix, accelerating oxidation and cross-linking. This leads to increased brittleness, suggesting that moisture acts as a catalyst for rheological degradation in hot, humid environments. The interaction depicted in Figure 8d underscores the cumulative nature of photo-oxidation. Increasing UV intensity promotes immediate surface hardening, which is further amplified over prolonged exposure times. The synergistic effect is particularly evident at high UV levels, where prolonged exposure leads to a sharp non-linear increase in G*, signaling severe embrittlement and a substantial loss of viscoelastic flexibility. Figure 8e shows that UV radiation and humidity jointly accelerate the formation of cross-linked molecular structures. While UV radiation drives the primary photochemical reactions, high humidity levels likely promote the formation of polar functional groups, further stiffening the material. A saturation point is observed at extreme levels, where G* stabilizes, indicating a maximum degree of aging-induced hardening under these combined stresses. The combined influence of aging time and humidity (Figure 8f) highlights the long-term impact of moisture on aging kinetics. Higher humidity promotes deeper oxygen penetration over time, resulting in a more pronounced increase in G* compared to dry aging conditions. Similar to the UV–humidity interaction, a threshold effect is observed followed by a plateau, suggesting that the material reaches oxidative saturation after prolonged exposure to moisture.

The response surface analysis reveals that the rheological evolution of the binder is not a simple additive result of individual environmental factors but a complex outcome of their synergistic coupling. The interplay between UV radiation, temperature, and moisture significantly shifts the aging kinetics, leading to accelerated stiffness gain and embrittlement that exceeds the effects of any single factor. These findings underscore that neglecting interactive effects would lead to a substantial underestimation of binder degradation in the field. Consequently, the established interaction models provide a more accurate framework for predicting the long-term performance of pavements, offering critical guidance for material selection and design optimization in regions exposed to extreme and multi-climatic stressors.

### 3.2. Impact of Environmental Factors on SBS-Modified Binder Performance

#### 3.2.1. Effect of Single-Factor Influences on SBS-Modified Binder Modulus in Natural Aging

The sensitivity analysis of the complex modulus provides critical insights into the rheological stability and degradation mechanisms of the SBS-modified binder under diverse aging conditions. Given the composite nature of the modified binder, its performance evolution is governed by the interplay between the elastomeric modifier and the bituminous matrix, leading to distinct response patterns compared to conventional binders. Figure 8 depicts the variations in G* across five coded experimental levels, serving as a basis for identifying the dominant aging drivers and their respective impact intensities.

**Figure 9:**
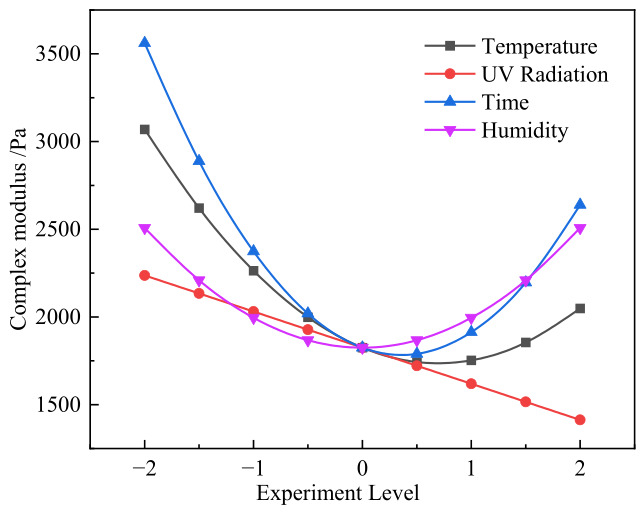
Effect of aging variables on the complex modulus of SBS-modified binder.

As illustrated in Figure 9, the complex modulus of the SBS-modified binder exhibits distinct non-linear response patterns to variations in temperature, UV radiation, aging time, and humidity across the coded experimental levels. Specifically, G* shows a continuous monotonic decrease with increasing UV radiation levels, suggesting that photo-induced polymer degradation is the predominant mechanism within the investigated range. In contrast, temperature, aging time, and humidity display a characteristic U-shaped trajectory; the modulus undergoes a sharp decline as experimental levels progress from −2 to 0, reaching a localized minimum at the center point, followed by a gradual recovery as levels advance toward +2. Among the variables, aging time and temperature exhibit the highest sensitivity in the low-level range, contributing to the most significant initial reductions in G*.

The complex shear modulus of the SBS-modified binder is significantly influenced by a combination of factors, including temperature, UV radiation, time, and humidity, each contributing to distinct degradation and hardening processes. Temperature and UV radiation primarily accelerate the degradation of the SBS modifier, leading to a substantial decrease in modulus. However, as the degradation process stabilizes, the binder’s hardening effect gradually becomes more pronounced, resulting in a modest recovery in modulus. Over time, oxidation and crosslinking reactions within the binder drive its gradual hardening, ultimately leading to an increase in modulus. Furthermore, humidity plays a key role in enhancing the hardening process, with a relatively smaller effect on SBS degradation. The inflection points observed in the experimental curves illustrate the dynamic interaction between SBS degradation and binder hardening, revealing the combined effects of both processes on the material’s long-term behavior and durability.

#### 3.2.2. Effect of Environmental Factor Interactions on SBS-Modified Binder Modulus

**Figure 10:**
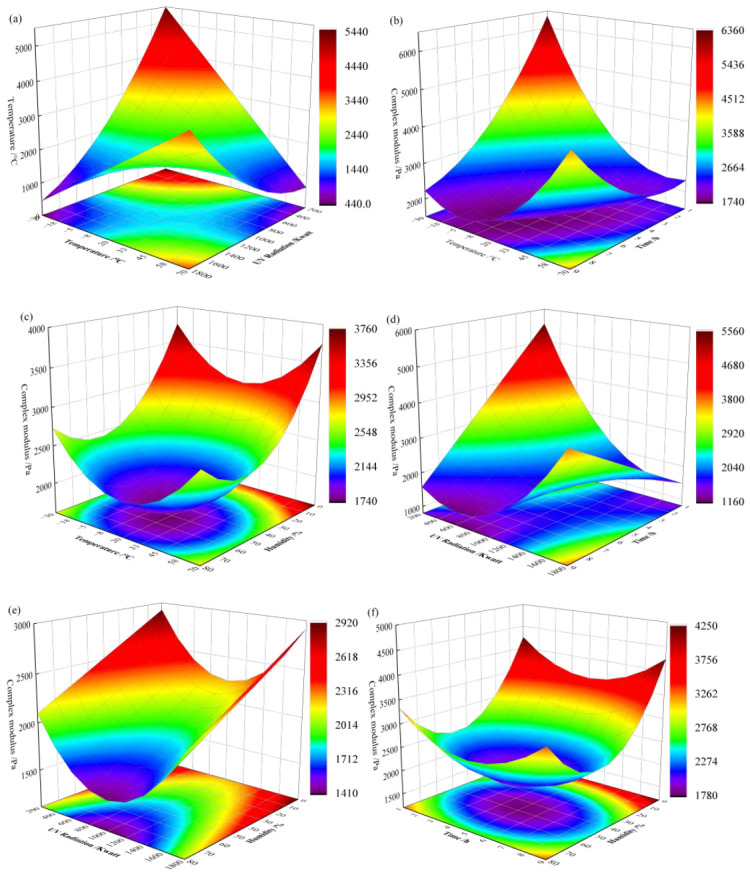
Effect of different experimental factors on the complex modulus of SBS asphalt. (**a**) Temparature-UV radiation; (**b**) Temparature-Time; (**c**) Temparature-Humidity; (**d**) UV radiation-Time; (**e**) UV radiation-Humidity; (**f**) Time-humidity.

The performance of SBS-modified binder is significantly influenced by environmental aging factors such as temperature, UV radiation, time, and humidity. These factors can lead to both the degradation of the SBS modifier and the hardening of the binder, which in turn affect the material’s complex shear modulus, a key indicator of its stiffness and resistance to deformation. Understanding the interaction between these environmental factors is essential for optimizing the durability and performance of SBS-modified binder in real-world applications, such as road construction. This study investigates the combined effects of temperature, UV radiation, time, and humidity on the complex shear modulus of SBS-modified binder under natural aging conditions, as shown in Figure 10.

The 3D response surface plots in Figure 9 illustrate the complex interactions between environmental factors and their impact on the G* of SBS-modified binder. Unlike the base binder, the rheological evolution of the SBS-modified binder is governed by the competition between polymer scission and bitumen oxidation. As shown in Figure 10a, G* undergoes a sharp reduction as both temperature and UV intensity increase. This trend indicates that elevated thermal and photo-stresses synergistically accelerate the degradation of the SBS polymer network, significantly reducing material stiffness. At extreme levels of both factors, the rate of decline stabilizes, suggesting a localized equilibrium where the onset of oxidative hardening in the binder matrix begins to counteract the softening caused by polymer scission. Figure 10b reveals an antagonistic relationship between temperature and exposure duration. While rising temperatures promote SBS degradation and an initial drop in G*, the influence of aging time facilitates a gradual recovery of the modulus. This reflects the time-dependent accumulation of oxidative products and cross-linking in the binder, which eventually offsets the softening effect of the degraded modifier. Figure 10c illustrates that humidity modulates the thermal response of the binder. While high temperatures drive polymer degradation, increased humidity promotes oxidative reactions that contribute to binder hardening. Consequently, G* decreases significantly under hot–dry conditions but shows a comparative increase at moderate temperatures and high humidity, indicating that moisture can act as a catalyst for binder stiffening. The coupling of UV radiation and aging time (Figure 10d) underscores a transition in the dominant aging mechanism. Rapid SBS degradation under intense UV exposure initially lowers G*; however, prolonged exposure time allows for sufficient binder oxidation to stabilize the modulus. This interplay suggests that the long-term stiffness of the material is a net result of early-stage polymer breakdown and late-stage matrix hardening. As depicted in Figure 10e, UV radiation and humidity exert opposing influences on the viscoelastic response. UV-induced photo-degradation primarily reduces G*, whereas humidity enhances the oxidative hardening process. At high levels of both stressors, the modulus stabilizes, reflecting a balance between the loss of polymer elasticity and the gain in binder viscosity. Figure 10f demonstrates that the combined effect of humidity and time significantly drives the hardening phase of the modified binder. While UV radiation remains the primary factor in polymer degradation, the synergistic effect of moisture and extended aging time accelerates the diffusion of oxygen and the subsequent formation of polar groups in the binder. This leads to a dynamic increase in stiffness, partially mitigating the rheological losses incurred by the breakdown of the SBS network.

In conclusion, the interactions between temperature, UV radiation, time, and humidity significantly affect the complex shear modulus of SBS-modified binder, highlighting the intricate balance between degradation and hardening processes. Temperature and UV radiation accelerate the degradation of the SBS modifier, resulting in a decrease in modulus, while time and humidity promote the hardening of the binder, leading to an eventual increase in modulus. The analysis reveals that at higher levels of temperature and UV radiation, the modulus decreases sharply, but this effect is mitigated over time, with hardening processes becoming more pronounced. Additionally, humidity counteracts the softening effects caused by temperature and UV radiation by promoting oxidation and crosslinking reactions. The dynamic interaction between these factors underscores the complexity of SBS-modified binder behavior under natural aging conditions. These findings emphasize the importance of considering all environmental factors simultaneously when evaluating the long-term performance and durability of SBS-modified binder in practical applications.

### 3.3. The Influence of Environmental Factors on CI and SI of Base Binder

#### Chemical Structure Evolution via FT-IR Analysis

**Figure 11:**
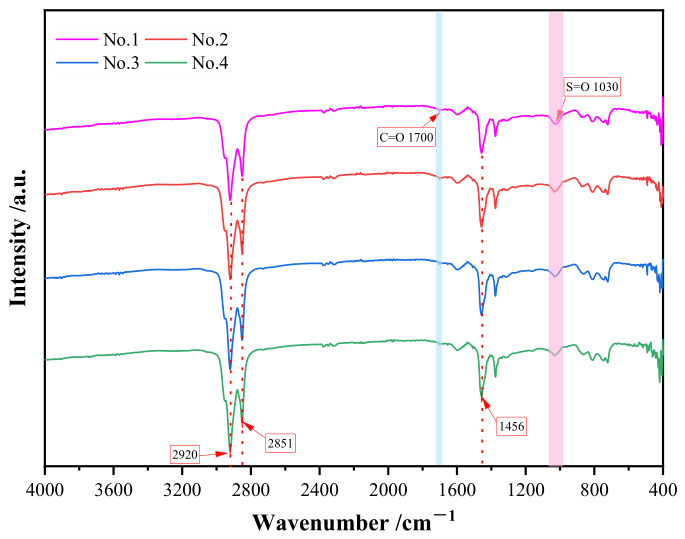
Infrared spectra of the base binder after aging at different horizons.

The structural transformation of the base binder under various aging protocols was further investigated using Fourier Transform Infrared (FT-IR) spectroscopy. FTIR measurements were performed on all indoor accelerated-aged samples (25 conditions in Table 2) as well as on the field-aged samples. The FT-IR spectra labeled No.1 to No.4 (as shown in Figure 11) were obtained from the corresponding experimental runs listed in Table 2. Specifically, No.1 represents the most severe aging condition where all four environmental factors were maintained at their high coded levels. No.2 through No.4 represent variations where humidity, aging time, or both were adjusted to their low coded levels, respectively, while maintaining high temperature and radiation intensity. This selection allows for a direct comparison of the individual and coupled effects of these stressors on the oxidative degradation of the binder.

As depicted in Figure 11, the infrared spectra provide a fingerprint of the chemical changes occurring within the bitumen matrix. The absorption bands at 2920 cm^−1^ and 2851 cm^−1^, attributed to the asymmetric and symmetric stretching vibrations of aliphatic C–H bonds in methyl and methylene groups, show negligible fluctuations in intensity across all aging conditions. Due to their relatively high stability during the oxidative process, these aliphatic peaks were utilized as internal standards for the quantitative calculation of structural indices, ensuring the comparability of the results.

The most significant chemical evolution is observed in the oxygenated functional group regions. The growth of the carbonyl peak (C=O) at approximately 1700 cm^−1^ and the sulfoxide peak (S=O) at 1030 cm^−1^ signify the progression of oxidative aging. From a mechanistic perspective, these changes are driven by free-radical chain reactions where environmental stressors—particularly thermal energy and UV radiation—initiate the cleavage of carbon chains and the subsequent capture of oxygen molecules. The sample under condition No.1 displays the highest peak area for both carbonyl and sulfoxide groups, as clearly highlighted in the inset. This suggests that the synergistic interaction of the aging factors in condition No.1 triggers a more aggressive oxidative pathway, leading to a higher concentration of ketone and carboxylic acid structures.

Quantitatively, the increase in the Carbonyl Index (C=O) and Sulfoxide Index (S=O) corresponds to a fundamental shift in the bitumen’s molecular composition. The formation of these strong polar functional groups increases the intermolecular associations via hydrogen bonding and dipole–dipole interactions. This molecular aggregation facilitates the transition of maltenes into asphaltenes, effectively increasing the asphaltene content and the colloidal instability of the binder. Macroscopically, this chemical restructuring manifests as the “aging hardening” effect, where the binder matrix exhibits an increased complex modulus and reduced phase angle, eventually leading to the loss of stress relaxation capacity and increased susceptibility to fatigue and low-temperature cracking.

### 3.4. The Influence of Environmental Factors on CI and SI of the SBS-Modified Binder

**Figure 12:**
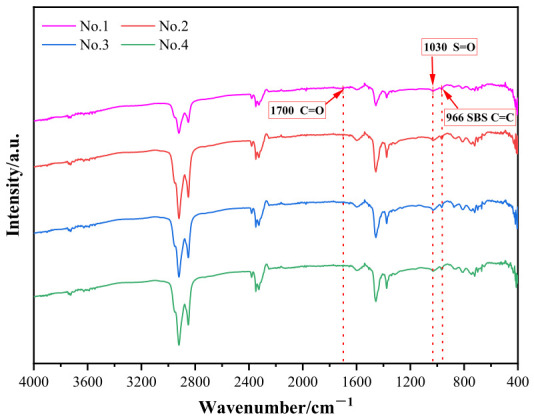
Infrared spectra of the SBS-modified binder after aging at different horizons.

The structural evolution of the SBS-modified binder under multi-factor coupled aging was characterized by the variations in its infrared spectra, as illustrated in Figure 12. Following the same selection logic as for the base binder (Section Chemical Structure Evolution via FT-IR Analysis), the spectra labeled No. 1 to No. 4 correspond to the experimental runs listed in Table 2: No. 1 represents the most severe aging condition (all four factors at high coded levels), while No. 2–No. 4 represent variations with low humidity and/or short aging time. The prominent intensification of the carbonyl (C=O) peak at approximately 1700 cm^−1^ and the sulfoxide (S=O) peak at 1030 cm^−1^, particularly in sample No. 1, signifies a profound degree of oxidative transformation within the binder matrix. This chemical restructuring is driven by high-energy environmental stressors—specifically the synergistic effect of UV radiation and thermal energy—which initiate free-radical chain reactions and the subsequent capture of oxygen molecules. The magnitude of these oxygenated functional groups serves as a direct indicator of aging severity, confirming that the coupling of multiple stressors accelerates the oxidation kinetics far more aggressively than any single factor acting in isolation.

Simultaneously, the structural integrity of the SBS modifier undergoes significant degradation, as evidenced by the progressive reduction in the characteristic peak at 966 cm^−1^. This peak, corresponding to the trans-CH bending vibration of the polybutadiene (PB) segment, serves as a “fingerprint” for the status of the elastomeric network within the binder. The decrease in peak intensity suggests the scission of long-chain polymer molecules into shorter fragments under the influence of photo-oxidation and thermal stress. This polymer degradation exerts a “softening” effect on the binder’s rheological profile, which partially counteracts the “hardening” effect caused by the oxidation of the bitumen matrix. This dual mechanism explains the non-linear sensitivity and the characteristic “U-shaped” evolution of the complex modulus observed in the macroscopic performance tests.

In contrast to the infrared spectra of the aged base binder, the SBS-modified binder exhibits a more complex chemical response due to the presence and subsequent degradation of the polymer-specific peaks. In the base binder, the aging process is typically characterized by a monotonic and simple increase in the carbonyl and sulfoxide indices, leading to a straightforward hardening effect. However, the spectra for the SBS-modified binder reveal a distinctive “competitive mechanism” between matrix oxidation and polymer scission. While the base binder primarily experiences the transition of maltenes into asphaltenes, the modified binder undergoes a simultaneous collapse of the elastomeric network. Consequently, the rheological evolution of the SBS-modified binder is not merely a result of increased viscosity but a delicate balance between the brittleness of the aged bitumen matrix and the loss of elasticity from the degraded SBS modifier, a feature that is entirely absent in conventional unmodified binders.

### 3.5. Evolution of Base Binder Modulus During the Natural Aging Process

**Figure 13:**
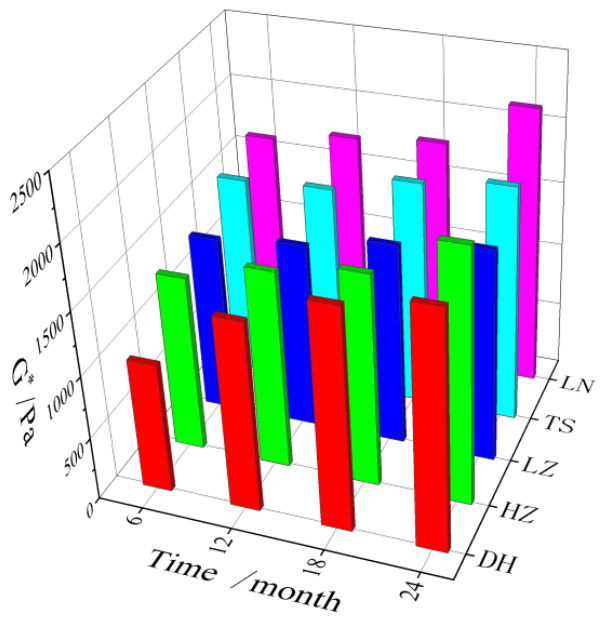
The relationship between the matrix binder modulus and time at different observation stations during the natural aging process.

The analysis of the modulus change in the base binder across different regions reveals notable differences driven by environmental factors, particularly moisture levels, temperature, and possibly UV radiation (Figure 13). In regions with higher moisture levels, the binder exhibits a more rapid increase in modulus over time, suggesting that increased moisture accelerates oxidation and crosslinking reactions, leading to a stiffer, more brittle material. Conversely, in drier regions, the modulus increases more gradually, reflecting slower oxidation and less aggressive hardening processes. Across the six regions, there is a consistent upward trend in the modulus, but the rate of increase varies. This suggests that each region’s environmental conditions are influencing the binder’s aging process differently. For instance, regions with a combination of high temperature and humidity likely accelerate aging due to the synergistic effects of these environmental factors. On the other hand, regions with lower humidity and possibly cooler conditions exhibit a more gradual increase in modulus, indicating slower oxidation and less crosslinking. This indicates that the binder in dry conditions undergoes a less intense aging process, potentially maintaining greater flexibility and durability. Furthermore, the graph highlights that regions with a combination of high humidity and temperature see a sharper rise in modulus, emphasizing the synergistic effect of moisture and heat in accelerating aging. These findings demonstrate how different environmental conditions play a critical role in the aging rate of the binder, with more humid areas experiencing faster and more pronounced hardening, while drier areas exhibit a slower aging process. This underscores the importance of considering regional environmental factors when designing asphalt mixtures to ensure long-term performance and durability. There may be a threshold point at which the modulus change accelerates significantly. This could occur when certain environmental conditions (e.g., high temperature combined with high humidity) surpass a critical level, pushing the binder into a more rapid aging process. This is likely reflected in the sharp rises observed in the graph as time progresses, especially for regions with high moisture levels.

The complex shear modulus (G*) measured at 60 °C and 1 rad/s of the binder obtained from various field observation sites demonstrates a consistent temporal pattern, which aligns closely with the relationship between G* at 60 °C and time derived from indoor multi-factor coupled aging orthogonal experiments. This consistency indicates that indoor accelerated aging simulations, based on multi-factor coupling, can effectively replicate the natural aging process of the binder. Additionally, through mathematical analysis, a corresponding relationship between the accelerated aging process in the laboratory and natural aging can be established. This finding suggests that multi-factor coupled aging simulations can be used as a reliable tool for modeling and predicting the long-term performance of the binder under natural aging conditions.

### 3.6. Evolution of SBS-Modified Binder Modulus During the Natural Aging Process

**Figure 14:**
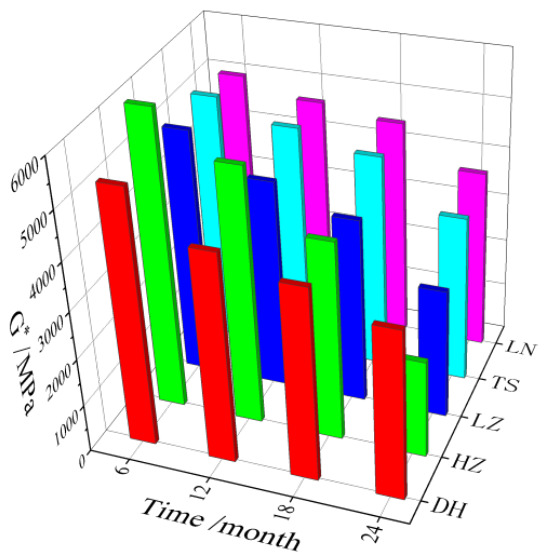
Relationship between the modulus and time of the SBS-modified binder at different observation sites during the natural aging process.

The analysis of the SBS-modified binder modulus across different regions indicates a consistent decline over time, which suggests a softening effect as the material ages (Figure 14). This reduction in modulus is primarily due to oxidative degradation, SBS polymer breakdown, and moisture-induced aging, all of which contribute to the decreasing stiffness of the binder. Environmental factors, including temperature, UV radiation, and humidity, play a significant role in accelerating this aging process. The combined effects of these factors lead to a more rapid decline in modulus, especially in regions exposed to higher temperatures and UV radiation. In areas where both temperature and UV radiation are high, the modulus decreases more sharply, indicating accelerated oxidative processes. The greatest reduction in modulus is observed in regions with high humidity, where moisture exacerbates oxidation and cross-linking, further decreasing the stiffness of the binder.

However, regional variations exist in the rate and magnitude of modulus reduction. In regions with milder temperatures or lower UV radiation intensity, the decrease in modulus occurs more gradually. In contrast, binder exposed to extreme environmental conditions, including high heat and UV radiation, experiences more significant aging and degradation, leading to a greater reduction in modulus. The synergistic effects of temperature, UV radiation, and aging time are especially evident in these regions, where both high temperatures and prolonged exposure to UV radiation accelerate the aging process, leading to faster cross-linking of the polymer chains and increased brittleness. Moisture further enhances these effects, accelerating the oxidative aging processes and leading to a stiffer material in regions with high humidity.

The aging mechanism of the SBS-modified binder involves the breakdown of the SBS modifier, which reduces the material’s elasticity and flexibility, making it more prone to cracking and deformation. The combined impact of temperature, UV radiation, and aging time results in a material that gradually loses its ability to resist deformation. The results indicate that local environmental conditions significantly influence the rate and extent of SBS-modified binder degradation. Therefore, understanding the interaction between these factors is critical for designing materials that can withstand varying environmental stresses and maintain long-term durability.

The G* at 60 °C and 1 rad/s of the SBS-modified binder obtained from different field observation sites is highly consistent with the relationship between G* at 60 °C and time derived from indoor multi-factor coupled aging orthogonal experiments (less than 6 h). This suggests that indoor accelerated aging simulations based on multi-factor coupling can effectively replicate the natural aging process of the SBS-modified binder. Furthermore, the corresponding relationship between indoor accelerated aging through multi-factor coupling and natural aging can be established using mathematical analysis methods.

### 3.7. Prediction of Natural Aging of Binder Based on Indoor Accelerated Aging

The Strategic Highway Research Program (SHRP) in the United States proposed the Asphalt Long-Term Aging Simulation (PAV) as one of its main research outcomes. The PAV test involves subjecting the binder to high temperature and high pressure in a sealed container for up to 20 h, simulating the aging of binder in pavements after 5 to 10 years of long-term aging. During the PAV aging process, except for regions with desert climates where the aging temperature is increased, the same aging parameters are used across all other areas. While standardized experimental parameters facilitate performance comparisons between different materials, significant differences in the aging process of the binder across regions with varying climatic conditions—such as temperature, UV radiation intensity, and humidity—suggest that the PAV simulation has limitations in accurately reflecting binder aging in diverse environments. The simulation or prediction of binder aging during service should closely align with local environmental factors, using specific environmental parameters as inputs for the simulation or prediction process. This study adopts an accelerated aging simulation method, incorporating temperature, UV radiation intensity, humidity, and other environmental parameters as factors in the aging process. By substituting the variables for temperature, UV radiation intensity, humidity, and the dependent variable shear modulus with the actual parameters obtained from a specific observation site at certain time points, a precise relationship can be established between binder aging in different climatic regions and accelerated aging time. This approach allows for an accurate simulation of binder aging in various regions based on their specific climate conditions. The results are shown in Table 4 and Table 5 for the base binder and the SBS-modified binder, respectively.

A quadratic polynomial regression model is used for regression analysis to express the mathematical relationship between the shear modulus of binder materials and experimental parameters such as temperature, radiation, time, and humidity. Based on a significance level of *p* > 0.1, the non-significant terms are removed, and the simplified regression equations for the base binder and SBS-modified binder are obtained, as shown in Equation (6) and Equation (7), respectively.

(6) $$Y_{B\mathrm{ase}}=553.37-3.67x_{1}+0.032x_{2}+56.19x_{3}-9.50x_{4}+0.0072x_{1}x_{2}-1.017x_{1}x_{3}-0.0025x_{1}x_{4}-0.0079x_{2}x_{3}+0.47x_{3}x_{4}$$

(7) $$Y_{SBS}=7330.58-115.41x_{1}-4.16x_{2}-905.23x_{3}+13.73x_{4}+0.048x_{1}x_{2}+7.78x_{1}x_{3}-0.037x_{1}x_{4}+0.52x_{2}x_{3}-1.23x_{3}x_{4}$$

where Y represents the dependent variable, specifically the mechanical modulus (MPa) of the binder, which serves as an indicator of its aging degradation. The terms $x_{1}$, $x_{2}$, $x_{3}$, and $x_{4}$ denote the independent variables, representing ambient temperature (°C), ultraviolet (UV) radiation intensity (W·m^−2^), relative humidity (%), and accelerated aging time (h), respectively.

Based on the natural aging condition parameters from Table 4 and Table 5 and the corresponding regression relationship between natural aging and indoor accelerated aging calculated using Equations (3) and (4), the results for different regions are summarized in Table 4 and Table 5. According to these results, when environmental parameters (temperature, humidity, radiation intensity) in the laboratory are aligned with those of the corresponding climate regions, the corresponding indoor aging time can be obtained. This enables the differential simulation of binder aging across various climate zones, ensuring the accuracy of the aging simulation. Taking the base binder in DH as an example, the binder’s natural aging period of 6 months corresponds to 13.2 h of indoor accelerated aging, 12 months corresponds to 21.5 h, 18 months corresponds to 26 h, and 24 months corresponds to 28.1 h of indoor accelerated aging. Taking SBS-modified binder in DH as an example, the natural aging period of 6 months corresponds to 0.6 h of indoor accelerated aging, 12 months corresponds to 1.9 h, 18 months corresponds to 2.4 h, and 24 months corresponds to 3.0 h of indoor aging.

## 4. Conclusions

Based on the multi-factor coupled aging experiments and field observations conducted in this study, the following primary conclusions can be drawn:

(1) The degradation of base binder is not merely an additive accumulation of individual environmental stressors but a highly synergistic process. While UV radiation and exposure time independently drive a monotonic increase in the complex modulus, the combination of intense UV, elevated temperatures, and high humidity acts as a powerful catalyst. This combined exposure accelerates oxygen diffusion, resulting in severe material embrittlement that single-factor tests fail to capture.

(2) Unlike the base binder, the SBS-modified binder exhibits a distinct, non-linear (U-shaped) rheological evolution resulting from a competitive aging mechanism. Initially, high temperatures and UV radiation severely degrade the elastomeric network, causing a rapid reduction in the complex modulus. However, over prolonged exposure and under the influence of humidity, the oxidative hardening of the bitumen matrix eventually outpaces the polymer degradation, leading to a gradual recovery in stiffness.

(3) FT-IR spectroscopy precisely maps the different aging pathways of the two binders. For the base binder, deep aging under coupled conditions drastically increases the Carbonyl Index (C=O) and Sulfoxide Index (S=O), driving the rapid transition of maltenes into asphaltenes. For SBS-modified binder, aging is characterized by the simultaneous oxidation of the matrix (rising C=O and S=O peaks) and the collapse of the elastomeric network, evidenced by the progressive decay of the characteristic polybutadiene peak at 966 cm^−1^.

(4) The application of a second-order orthogonal regression composite design successfully established a robust mathematical bridge between indoor accelerated aging and natural field aging. By inputting actual local climatic variables (temperature, UV intensity, humidity, and time), the derived quadratic polynomial regression models can accurately calculate the equivalent indoor aging time required to match the field degradation of specific regions. This methodological advancement overcomes the limitations of standardized PAV testing, enabling accurate, climate-customized lifespan predictions for asphalt pavements worldwide.

## Figures and Tables

**Table 1:** Technical properties of the 90# base binder and SBS-modified asphalt.

Property	Unit	Base Asphalt	SBS-Modified Asphalt	Test Method
Penetration (25 °C, 5 s, 100 g)	0.1 mm	95	72	T0604
Penetration index (PI)	/	−0.75	0.41	T0604
Softening point (T&B)	°C	45.6	78.3	T0606
Ductility (10 °C)	cm	92	52 (5 °C)	T0605
Viscosity (60 °C)	Pa·s	168	890	T0620
Solubility	%	99.73	98.96	T0607
TFOT				
Mass change	%	−0.55	−0.28	T0609/T0610
Retained penetration ratio	%	58	77	T0604
Retained ductility (10 °C)	cm	9.3	22.5	T0605

**Table 2:** Definition of factors in the second-order orthogonal experimental design.

Factor	Zero Level	Variation Range	Low Level	High Level
Temperature	20 °C	25 °C	−5 °C	45 °C
Radiation Intensity	1000 watts	400 watts	600 watts	1400 watts
Time	5 h	2 h	3 h	7 h
Humidity	40%	20%	20%	60%

**Table 3:** Results of the experimental design for multi-factor aging simulation of binders.

No.	c1	c2	c3	c4	Temperature /°C	Radiation Intensity /Watts	Time /h	Humidity /%
1	1	1	1	1	45	1400	7	60
2	1	1	1	−1	45	1400	7	20
3	1	1	−1	1	45	1400	3	60
4	1	1	−1	−1	45	1400	3	20
5	1	−1	1	1	45	600	7	60
6	1	−1	1	−1	45	600	7	20
7	1	−1	−1	1	45	600	3	60
8	1	−1	−1	−1	45	600	3	20
9	−1	1	1	1	−5	1400	7	60
10	−1	1	1	−1	−5	1400	7	20
11	−1	1	−1	1	−5	1400	3	60
12	−1	1	−1	−1	−5	1400	3	20
13	−1	−1	1	1	−5	600	7	60
14	−1	−1	1	−1	−5	600	7	20
15	−1	−1	−1	1	−5	600	3	60
16	−1	−1	−1	−1	−5	600	3	20
17	−2	0	0	0	−30	1000	5	40
18	2	0	0	0	70	1000	5	40
19	0	−2	0	0	20	200	5	40
20	0	2	0	0	20	1800	5	40
21	0	0	−2	0	20	1000	1	40
22	0	0	2	0	20	1000	9	40
23	0	0	0	−2	20	1000	5	0
24	0	0	0	2	20	1000	5	80
25	0	0	0	0	20	1000	5	40

Note: c1, c2, c3, and c4 are the coded levels for temperature, radiation intensity, time, and humidity, respectively. A coded level of +1 corresponds to the high level of a factor, –1 corresponds to the low level, and 0 corresponds to the zero level. The actual values corresponding to these coded levels are given in Table 1. For example, for temperature, a coded level of +1 represents 45 °C, −1 represents −5 °C, and 0 represents 20 °C.

**Table 4:** The corresponding time relationship between natural aging matrix binder and indoor accelerated aging binder.

No.	Region	Observation Time/m	Modulus/Pa	Temperature/°C	UV Intensity/W·m^−2^	Humidity/%	Time/h
1	DH	6	1029.58	10.52	184.86	25.69	13.2
2		12	1498.62				21.5
3		18	1745.93				26.0
4		24	1864.21				28.1
5	HZ	6	1389.62	1.51	172.05	60.39	17.2
6		12	1562.57				19.4
7		18	1668.96				20.7
8		24	1993.85				24.6
9	LZ	6	1397.96	10.73	164.34	38.43	19.9
10		12	1462.01				20.9
11		18	1584.25				22.9
12		24	1665.38				24.2
13	TS	6	1590.35	10.42	67.54	62.29	22.3
14		12	1623.56				22.8
15		18	1777.36				24.9
16		24	1857.25				25.9
17	NL	6	1668.73	14.37	205.3	51.95	25.4
18		12	1765.23				26.9
19		18	1823.02				27.8
20		24	2180.84				33.4

**Table 5:** The corresponding time relationship between natural aging SBS binder and indoor accelerated aging binder.

No.	Region	Observation Time/m	Modulus/ Pa	Temperature/°C	UV Intensity/W·m^−2^	Humidity/%	Time/h
1	DH	6	5321.21	10.52	184.86	25.69	0.6
2		12	4358.96				1.9
3		18	3994.67				2.4
4		24	3500.52				3.0
5	HZ	6	6171.87	1.51	172.05	60.39	1.3
6		12	5320.16				2.2
7		18	4131.32				3.6
8		24	2026.18				6.0
9	LZ	6	5132.61	10.73	164.34	38.43	1.1
10		12	4356.21				2.1
11		18	3851.03				2.7
12		24	2694.53				1.7
13	TS	6	5209.35	10.42	67.54	62.29	1.7
14		12	4806.23				2.2
15		18	4468.88				2.6
16		24	3473.06				3.7
17	LN	6	5067.95	14.37	205.3	51.95	0.8
18		12	4736.32				1.2
19		18	4533.18				1.5
20		24	3736.20				2.5

## Data Availability

The original contributions presented in this study are included in the article. Further inquiries can be directed to the corresponding author.
